# A dignitary medicine curriculum developed using a modified Delphi methodology

**DOI:** 10.1186/s12245-020-00270-4

**Published:** 2020-02-21

**Authors:** Mobarak A. Al Mulhim, Robert G. Darling, Ritu Sarin, Alex Hart, Hetaf Kamal, Abdullah Al Hadhirah, Amalia Voskanyan, Lewis Hofmann, Bradley A. Connor, Roger A. Band, James Jones, Richard Tubb, Ronny Jackson, Amado Alejandro Baez, Edward Wasser, Sean Conley, William Lang, Gregory Ciottone

**Affiliations:** 1 Beth Israel Deaconess Medical Center, Harvard Medical School, Boston, USA; 2 Royal Clinics, Riyadh, Saudi Arabia; 3 Patronus Medical, LLC, Mountain View, USA; 4 Johns Hopkins Aramco, Dhahran, Saudi Arabia; 5 White House Physician Emeritus, Shoreland, Washington, D.C., USA; 6 The New York Presbyterian Hospital, Cornell Campus, New York, USA; 7 Sidney Kimmel Medical College, Thomas Jefferson University, Philadelphia, USA; 8 Medical Evaluation and Treatment Unit, WHMU, Washington D.C., USA; 9 The White House Medical Unit (WHMU), Washington, D.C., USA; 10 Department of Emergency Medicine, Medical College of Georgia, Augusta University, Augusta, USA; 11 Canada Protective Detail (PMPD), Royal Canadian Mounted Police (RCMP), Ottawa, Canada; 12 International Medicine, Inova Health System, Merrifield, USA

**Keywords:** Dignitary medicine, Curriculum, Curriculum development, Delphi methodology

## Abstract

**Background:**

Dignitary medicine is an emerging field of training that involves the specialized care of diplomats, heads of state, and other high-ranking officials. In an effort to provide guidance on training in this nascent field, we convened a panel of experts in dignitary medicine and using the Delphi methodology, created a consensus curriculum for training in dignitary medicine.

**Methods:**

A three-round Delphi consensus process was performed with 42 experts in the field of dignitary medicine. Predetermined scores were required for an aspect of the curriculum to advance to the next round. The scores on the final round were used to determine the components of the curriculum. Scores below the threshold to advance were dropped in the subsequent round.

**Results:**

Our panel had a high degree of agreement on the required skills needed to practice dignitary medicine, with active practice in a provider’s baseline specialty, current board certification, and skills in emergency care and resuscitation being the highest rated skills dignitary medicine physicians need. Skills related to vascular and emergency ultrasound and quality improvement were rated the lowest in the Delphi analysis. No skills were dropped from consideration.

**Conclusions:**

The results of our work can form the basis of formal fellowship training, continuing medical education, and publications in the field of dignitary medicine. It is clear that active medical practice and knowledge of resuscitation and emergency care are critical skills in this field, making emergency medicine physicians well suited to practicing dignitary medicine.

## Introduction

Dignitaries include heads of state, presidents, royal family members, government officials, ambassadors, celebrities, athletes, high-ranking business officials, and other very important persons (VIPs). Dignitaries have a unique set of healthcare needs [1], different than the general public, including the need for preventive and protective medical care, 24-h high-quality personalized healthcare, and an even greater degree of privacy than ordinary citizens. The power, prestige, and station that a dignitary has may result in providers being awestruck and providing dignitaries with sub-optimal care [2–4]. In addition, dignitaries are commonly surrounded by an inner circle of important family members and advisors who can hinder the doctor-patient relationship [5–8].

Dignitary medicine (DM) is often provided by government-appointed officials (such as the White House Medical Unit in the United States or the Royal Physicians in Saudi Arabia), consulting groups of self-identified experts, clinics marketed toward VIPs (Cleveland Clinic, Mayo Clinic, etc.), or by private concierge doctors [9].These providers often come from a variety of backgrounds, with very little standardization in their training or backgrounds.

To offer optimal medical services for dignitaries, as opposed to a fragmented approach delivered by a slew of specialists, we believe a formal curriculum is needed in the field of DM. This can serve as the basis of conferences and other continuing education modules or even fellowship training. In order to create an appropriate curriculum, the Delphi methodology has been used to develop a consensus-driven curriculum for medical training in a variety of fields [10–14].We sought to create an expert-driven consensus curriculum in DM using a modified Delphi methodology and present the results herein.

## Methods

Investigators used a modified, three-round Delphi methodology to create a curriculum in DM. The sample curriculum was drafted after performing a literature review for peer-reviewed articles on DM in PubMed (National Center for Biotechnology Information, National Institutes of Health; Bethesda, MD, USA) and Hollis (Harvard University; Cambridge, MA, USA). The authors held multiple discussions with experts in the field, and they ultimately drafted a curriculum that was used for the Delphi analysis.

A total of 42 recognized experts in the field of DM participated in the Delphi analysis. All providers had at least 3 years of experience in DM and were actively involved in the field at the time of the survey. Consent was required for the survey and obtained via e-mail. The Delphi analysis was performed via an online survey, using a Likert scale of one (not a priority) to five (essential priority) for each competency.

A three-round Delphi analysis was utilized, with all data recorded in Beth Israel Deaconess’s Institutional RedCap database (Research Electronic Data Capture, Vanderbilt University). Descriptive statistics were tabulated in RedCap. In each round, respondents rated each category and individual competencies within each category. A pre-defined average score of 3 or less was used to exclude any item from future rounds. Respondents were blinded to individual respondent’s scores, but did see the result of all aggregate scores for every category at the beginning of rounds 2 and 3. Each subsequent round was based upon whichever items remained from the prior round.

In addition, all participants had demographic information collected via RedCap. They also were invited to answer a brief survey regarding their experience and training related to DM. Those data are presented, along with the results of the Delphi analysis.

## Results

A total of 42 respondents from 12 different countries answered the survey and all respondents answered each question in all rounds. The average age of the respondents was 52 ± 9, with a range of 34–70. Experience with DM was extensive, with only 5/42 (12%) of respondents having fewer than 5 years in the field and 30/42 (71%) having over 10 years of experience. Most respondents had government security clearance when they were on assignment (32/42, 76%). Among the respondents, 69% felt prepared for their DM assignment; however, 93% felt specialized training would benefit other physicians interested in DM. Of all respondents, 83% maintain at least a part-time clinical practice away from DM and all respondents felt at least 25% of a DM physician’s time must be spent in active practice to maintain competence and clinical skills.

The complete list of categories and competencies with the final Likert score on the third round of the Delphi analysis can be seen in supplementary Appendix 1. There were no items that were removed from consideration in any round of the survey, as no item had an average score less than 3. Furthermore, no items had a change in average score between any of the rounds. There are six broad themes that were surveyed and multiple competencies within each category, which are listed in Table 1. The six themes are executive health, protective medicine, clinical competence, wellness and longevity, advances in medical technology, and leadership.

**Table 1:** Broad skill areas identified in the survey

Category	Specific skills
1- Executive health	Integrating the dignitary into the medical system
	Concierge medical practice skills
	Written care plans
	Coordination of care at home and while traveling
	Attention to the unique psyche and needs of dignitaries
	Being in proximity to the dignitary
2- Protective	Basic EM and disaster skills
	Threat assessment
	Disaster training and drills
	Security umbrella
	Medical evacuation
	Motorcade operations
	Design and upkeep of medical kits and go bags
	Medical risk and threat assessment
Clinical competency	Maintenance of clinical skills
	Active medical practice
	Active certifications
	Continuing medical education
Wellness and longevity	General wellness program
	Understand guideline-based preventive care
	Personalized care plan for the patient
	Preventive care, such as vaccines and travel medicine
Leadership	Maintain certifications for all staff
	Communication skills with the media
	Crisis management
	Team management
Technology	Electronic record keeping and security
	Wearables
	Point of care diagnostics

The highest rated individual skills in the entire survey were maintenance of medical skills and certification, along with having adequate skills in basic evaluation and resuscitation. This is further described in Table 2. The lowest rated sub-competencies included developing a precision medical plan for a client’s unique needs, understanding wearable technologies, being able to perform emergency ultrasound procedures(including eFAST and obtaining vascular access), and being able to execute quality improvement projects and monitor these metrics. Table 3 further lists the lowest rated skills. The full details of all questions asked in the survey and scores for the third round of questions are available in supplementary Appendix 1. Specific knowledge, skills, and abilities (KSAs) are listed in Table 4 as well.

**Table 2:** Highest rated competencies

Maintain skills and medical specialty certification	**4.9**
Demonstrate competency in basic essential emergency medical care and resuscitation	**4.8**

**Table 3:** Lowest rated competencies

Perform eFAST and vascular bedside ultrasound	4.1
Demonstrate KSAs in the use of virtual medicine technologies in the delivery of high-quality patient care	4.1
Demonstrate knowledge and skills in the utilization of telemonitoring and wearable technology	4.1
Demonstrate knowledge and skills in the metrics of health care system quality (CQM) and outcomes	4.0
Develop precision personalized medical care and wellness plans based on a client’s unique needs	**3.9**

**Table 4:** Knowledge, skills, and abilities identified in dignitary medicine curriculum

Skill area	Knowledge, skills, and abilities (KSAs)
Protective medicine	Demonstrate an understanding of KSAs in basic Disaster Medicine and Emergency Management
Protective medicine	Demonstrate an understanding of the KSAs of implementing an integrated emergency medical service system into the security umbrella surrounding the client
Protective medicine	Demonstrate an understanding of KSAs in the processes and procedures of medical evacuation from the most likely scenarios
Protective medicine	Demonstrate an understanding of KSAs in motorcade operation procedures
Protective medicine	Demonstrate an understanding of KSAs in the application of medical intelligence in risk assessment in medical operations
Protective medicine	Demonstrate an understanding of KSAs in public relations including media training
Clinical competency	Demonstrate an understanding of KSAs in basic essential emergency medical care and resuscitation
Clinical competency	Demonstrate an understanding of KSAs in trauma field care
Advances in medical technology and electronic records	Demonstrate KSAs in the use of telemedicine technologies in the delivery of high-quality patient care
Advances in medical technology and electronic records	Demonstrate KSAs in the integration and utilization of advanced telecommunications in patient care
Advances in medical technology and electronic records	Demonstrate KSAs in the use of the EMR while strictly following security and patient confidentiality
Advances in medical technology and electronic records	Demonstrate KSAs in the use of cutting-edge point of care diagnostic testing devices
Leadership	Demonstrate KSAs in crisis and leadership management
Leadership	Demonstrate KSAs in medical unit governance and operation
Leadership	Demonstrate KSAs in strategic planning and the importance of team work
Leadership	Demonstrate KSAs in medical unit design and setup (hospital-based, mobile, and in-residence)
Leadership	Demonstrate KSAs in the metrics of health care system quality and outcomes
Leadership	Demonstrate KSAs in medical intelligence interpretation and data base analysis to include identifying centers of medical excellence and medical experts from any region of the world

## Discussion

We utilized a three-round Delphi methodology to query 42 experts in DM regarding critical topics for a consensus curriculum in DM. Those surveyed had a strong agreement on the broad themes of executive health, protective medicine, clinical competence, wellness of the dignitary, medical technology/electronic records, and leadership. Furthermore, there was extensive agreement on the specific competencies within the curriculum. Agreement was so profound that no item was discarded during any of the three rounds of the Delphi analysis. Such a high degree of agreement lends substantial weight to our suggested curriculum.

There are both broad themes in DM training that were endorsed by our survey and specific skills that are critical to the DM physician. At a high level, it is clear that DM physicians must possess unique skills that allow them to interact with dignitaries on a routine basis, in their living quarters, and be aware of both their need for privacy and their unique psychology. Furthermore, it is clear that the DM physician needs a broad array of medical skills in the outpatient, inpatient, and emergency settings [15–19]. Finally, the DM physician needs to be able to create protocols and integrate the care of the VIP into the existing infrastructure of our health care system.

Based on our analysis, the critical skills required to practice DM include executive healthcare, protective medicine, clinical competence, wellness, advances in medical technology, and leadership. Executive health includes concierge medical skills and understanding the unique needs of the dignitary physician relationship, especially the dignitary’s need for privacy. Protective medicine involves tactical, travel, and disaster-related medical skills. It also includes coordinating care with security forces and other teams involved with the dignitary. Clinical competence refers to maintenance of skills and ongoing board certification in a DM physician’s base specialty. Wellness focuses on screening, preventive medicine, and the best care for chronic illness in the dignitary. Emphasis on personalized medicine and guideline-based practice/clinical key performance indicators (KPIs) is critical. Advances in medical technology include the dignitary physician being up-to-date on technologic resources, electronic medical record, cybersecurity, and the role of telemedicine in providing timely consultation from anywhere in the world. Leadership involves the skills needed to coordinate and orchestrate care for the dignitary, including managing the entourage around the dignitary.

Although most of our respondents felt that the specific skills we laid out in the curriculum are important, there were a few areas that stood out as being either critical or optional. On the critical side, providers clearly must be board certified practitioners with appropriately up-to-date credentials and engaged in active practice and continuing education. It is clear that physicians without a strong ongoing clinical practice may not be appropriate providers in the field of DM. Furthermore, they must have competency and skills in the basics of emergency management and resuscitation. This may make DM a particularly appealing specialty for providers in specialties such as internal medicine, emergency medicine, family medicine, critical care, general surgery, and trauma care. Knowledge of women’s health and pediatrics will likely also be needed.

Areas where agreement was less common and skills that were less critical encompassed several areas. Specifically, knowledge of quality improvement was not seen as essential. Skills in telehealth, telemedicine, and wearable technology were also not seen as critical. Likewise, emergency ultrasound skills were not seen as integral to the DM physician curriculum. Nonetheless, even these areas of lesser agreement still generally scored a 4/5 on the Likert scale, which may still support their inclusion within the curriculum.

There are some limitations to our study. The respondents encompass a relatively tight-knit community and perhaps outside respondents may have differing opinions on the curriculum. There were no changes to any average rating for any question in any round, which brings into question whether a different subset of providers may have different opinions on the topics addressed or if the choice of initial topics was too narrow, inappropriate, or otherwise compromised. Furthermore, we did not ask respondents in an open-ended manner what they wanted in the curriculum; we presented curricular offerings to them and they voted as to whether they felt these were relevant. Perhaps other topics may have emerged if the respondents had more freedom to respond in an open manner to what they wanted to see in a DM curriculum. Given the limited number of respondents, other ideas may emerge with a broader population in the survey.

## Conclusion

We have identified a set of critical individual skills for providers in DM. Furthermore, we believe this can serve as an excellent template for continuing education in DM and possibly even formal fellowship training in DM. We believe physicians who practice emergency medicine, internal medicine, family medicine, tropical/travel medicine, general and trauma surgery, and critical care may be excellent candidates to practice DM.

## Supplementary information

**Additional file 1.** Appendix 1: Full Survey Results and Scoring from All Rounds of Analysis.

## Data Availability

All data is included in the paper, tables, and appendices. The initial survey responses are stored in the BIDMC RedCap Database; however, all data is published in the paper.
