# Supplementary material for: A dignitary medicine curriculum developed using a modified Delphi methodology
Source: Int J Emerg Med. 2020 Feb 21;13:11. doi: 10.1186/s12245-020-00270-4 (PMC7035733; doi:10.1186/s12245-020-00270-4)
Supplement: Supplementary file 1 — Additional file 1. Appendix 1: Full Survey Results and Scoring from All Rounds of Analysis. [file 12245_2020_270_MOESM1_ESM.docx]

**Appendix1: Full Survey Results and Scoring from All Rounds of Analysis**

| **Question** | **Round 1 Score** | **Round 2 Score** | **Final Round Score** | **Top Rating (%)** | **Rank** |
| --- | --- | --- | --- | --- | --- |
| ***1. Executive Health*** |  |  |  |  |  |
| **Demonstrate an understanding of how to integrate a client into a healthcare organization** | **4.5** | **4.5** | **4.5** | **60** | **10** |
| **Demonstrate an understanding of the care of the client in the Emergency Department, outpatient, and inpatient settings** | **4.6** | **4.6** | **4.6** | **76** | **6** |
| **Demonstrate proficiency in writing executive healthcare policies, protocols and procedures into healthcare plans geared toward the dignitary or other “special” client** | **4.2** | **4.2** | **4.2** | **29** | **20** |
| **Demonstrate an understanding of the unique ethical issues as they relate to executive healthcare** | **4.5** | **4.5** | **4.5** | **69** | **8** |
| **Demonstrate an understanding of the importance of confidentiality and privacy in caring for the client** | **4.8** | **4.8** | **4.8** | **88** | **2** |
| **Demonstrate an understanding of the importance of the effect of a client’s unique cultural differences on the ability to provide care** | **4.3** | **4.3** | **4.3** | **43** | **15** |
| **Demonstrate an understanding of the unique psychology and behavior of high-profile figures and how this may impact their health care** | **4.6** | **4.6** | **4.6** | **81** | **4** |
| **Understand the challenges of balancing direct and frequent access to the client while respecting the client’s need for privacy.** | **4.7** | **4.7** | **4.7** | **91** | **1** |
|  |  |  |  |  |  |
| *2. Protective Medicine* |  |  |  |  |  |
|  |  |  |  |  |  |
| **Demonstrate an understanding of KSA’s in basic Disaster Medicine and Emergency Management** | **4.5** | **4.5** | **4.5** | **50** | **13** |
| **Perform a hazard vulnerability analysis and medical threat assessment** | **4.7** | **4.7** | **4.7** | **74** | **7** |
| **Demonstrate an understanding of SKAs in the design and implementation of emergency and Disaster Medicine drill scenarios** | **4.4** | **4.4** | **4.4** | **45** | **14** |
| **Demonstrate an understanding of the KSAs of implementing an integrated emergency medical service system into the security umbrella surrounding the client** | **4.6** | **4.6** | **4.6** | **62** | **9** |
| **Demonstrate an understanding of KSAs in the processes and procedures of medical evacuation from the most likely scenarios** | **4.6** | **4.6** | **4.6** | **81** | **4** |
| **Demonstrate an understanding of KSAs in motorcade operation procedures** | **4.6** | **4.6** | **4.6** | **62** | **9** |
| **Design general emergency medical kits (GO Bags) and kits specific to the client’s unique medical requirements** | **4.6** | **4.6** | **4.6** | **86** | **3** |
| **Perform a comprehensive medical risk assessment plan** | **4.6** | **4.6** | **4.6** | **81** | **4** |
| **Demonstrate an understanding of KSAs in the application of medical intelligence in risk assessment in medical operations** | **4.5** | **4.5** | **4.5** | **57** | **11** |
| **Demonstrate an understanding of KSAs in public relations including media training** | **4.4** | **4.4** | **4.4** | **41** | **16** |
|  |  |  |  |  |  |
| **3.** *Wellness and Longevity* |  |  |  |  |  |
|  |  |  |  |  |  |
| **Review and assess currently available, state of the art Health, Wellness and Longevity programs and their appropriateness for the client** | **4.4** | **4.4** | **4.4** | **43** | **15** |
| **Manage and implement evidence-based lifestyle modification programs** | **4.4** | **4.4** | **4.4** | **33** | **18** |
| **Develop precision personalized medical care and wellness plans based upon client’s unique needs** | **4** | **4** | **4** | **26** | **21** |
| **Implement preventive medicine practices to include appropriate vaccinations** | **4.7** | **4.7** | **4.7** | **69** | **8** |
| **Develop a personalized screening program tailored to the client’s needs** | **4.5** | **4.5** | **4.5** | **55** | **12** |
| **Develop disease specific health care plans with outcome measures and carefully manage expectations** | **4.3** | **4.3** | **4.3** | **36** | **17** |
| **Demonstrate skill in the ability to council the client against the use of non-evidence-based treatments and potentially harmful alternative medicine practices** | **4.4** | **4.4** | **4.4** | **45** | **14** |
|  |  |  |  |  |  |
| **4.** *Clinical Competency* |  |  |  |  |  |
|  |  |  |  |  |  |
| **Maintain skills and medical specialty certification** | **4.9** | **4.9** | **4.9** | **88** | **2** |
| **Demonstrate an understanding of KSAs in basic essential emergency medical care and resuscitation** | **4.8** | **4.8** | **4.8** | **88** | **2** |
| **Demonstrate an understanding of KSAs in trauma field care** | **4.6** | **4.6** | **4.6** | **76** | **6** |
| **Perform e-FAST bedside ultrasound** | **4.1** | **4.1** | **4.1** | **21** | **22** |
|  |  |  |  |  |  |
| **5.** *Medical Technology* |  |  |  |  |  |
|  |  |  |  |  |  |
| **Demonstrate KSA’s in the use of telemedicine technologies in the delivery of high-quality patient care** | **4.1** | **4.1** | **4.1** | **29** | **20** |
| **Demonstrate KSA’s in the integration and utilization of advanced telecommunications in patient care** | **4.1** | **4.1** | **4.1** | **21** | **22** |
| **Demonstrate KSA’s in the use of the EMR while strictly following security and patient confidentiality** | **4.4** | **4.4** | **4.4** | **41** | **16** |
| **Demonstrate KSA’s in the use of cutting-edge point of care diagnostic testing devices** | **4.4** | **4.4** | **4.4** | **26** | **21** |
|  |  |  |  |  |  |
| *6. Leadership* |  |  |  |  |  |
|  |  |  |  |  |  |
| **Demonstrate KSA’s in crisis and leadership management** | **4.6** | **4.6** | **4.6** | **79** | **5** |
| **Demonstrate KSA’s in medical unit governance and operation** | **4.3** | **4.3** | **4.3** | **29** | **20** |
| **Demonstrate KSA’s in strategic planning and the importance of team work** | **4.3** | **4.3** | **4.3** | **31** | **19** |
| **Demonstrate KSA’s in medical unit design and set up (hospital-based, mobile and in- residence)** | **4.4** | **4.4** | **4.4** | **45** | **14** |
| **Demonstrate KSA’s in the metrics of health care system quality and outcomes** | **4** | **4** | **4** | **26** | **21** |
| **Demonstrate KSA’s in medical intelligence interpretation and data base analysis to include identifying centers of medical excellence and medical experts from any region of the world** | **4.6** | **4.6** | **4.6** | **79** | **5** |
|  |  |  |  |  |  |
